# Process evaluation of a tailored mobile health intervention aiming to reduce fatigue in airline pilots

**DOI:** 10.1186/s12889-016-3572-1

**Published:** 2016-08-26

**Authors:** Alwin van Drongelen, Cécile R. L. Boot, Hynek Hlobil, Tjabe Smid, Allard J. van der Beek

**Affiliations:** 1 Department of Public and Occupational Health, EMGO Institute for Health and Care Research, VU University Medical Center, PO Box 7057, 1007 MB Amsterdam, The Netherlands; 2 KLM Health Services, Schiphol Airport, Schiphol, The Netherlands; 3 Body@Work TNO VUmc, Research Center on Physical Activity, Work and Health, VU University Medical Center, Amsterdam, The Netherlands

**Keywords:** Work schedule tolerance, Primary prevention, Telemedicine, Implementation, Mobile health, Process evaluation

## Abstract

**Background:**

MORE Energy is a mobile health intervention which aims to reduce fatigue and improve health in airline pilots. The primary objective of this process evaluation was to assess the reach, dose delivered, compliance, fidelity, barriers and facilitators, and satisfaction of the intervention. The second objective was to investigate the associations of adherence to the intervention with compliance and with participant satisfaction. Thirdly, we investigated differences between the subgroups within the target population.

**Methods:**

The intervention consisted of a smartphone application, supported by a website. It provided advice on optimal light exposure, sleep, nutrition, and physical activity, tailored to flight and personal characteristics. The reach of the intervention was determined by comparing the intervention group participants and the airline pilots who did not participate. The dose delivered was defined as the total number of participants that was sent an instruction email. Objective compliance was measured through the Control Management System of the application. To determine the fidelity, an extensive log was kept throughout the intervention period. Subjective compliance, satisfaction, barriers, facilitators, and adherence were assessed using online questionnaires. Associations between the extent to which the participants applied the advice in daily life (adherence), compliance, and satisfaction were analysed as well. Finally, outcomes of participants of different age groups and haul types were compared.

**Results:**

A total of 2222 pilots were made aware of the study. From this group, 502 pilots met the inclusion criteria and did agree to participate. The reach of the study proved to be 22 % and the dose delivered was 99 %. The included pilots were randomized into the intervention group (*n* = 251) or the control group (*n* = 251). Of the intervention group participants, 81 % consulted any advice, while 17 % did this during four weeks or more. Fidelity was 67 %. The participants rated the intervention with a 6.4 (SD 1.6). Adherence was not associated with compliance, but was associated with satisfaction (*p* ≤ 0.001). Pilots of 35 to 45 year old were significantly more interested in advice regarding physical activity than their colleagues, and short-haul pilots were more interested in advice regarding nutrition compared to long-haul pilots.

**Conclusions:**

The MORE Energy intervention was well received, resulting in an adequate reach and a high dose delivered. The compliance and satisfaction scores indicate that engagement and functionality should be enhanced, and the content and applicability of the advices should be improved to appeal all subgroups of the target population.

**Trial registration:**

Nederlands Trial Register NTR2722. Registered 27 January 2011.

**Electronic supplementary material:**

The online version of this article (doi:10.1186/s12889-016-3572-1) contains supplementary material, which is available to authorized users.

## Background

Due to disruption of the sleep wake pattern and the circadian rhythm, fatigue is inevitable in occupations where individuals are required to work when they normally would be asleep [1]. In the aviation industry, fatigue management strategies have been developed to minimize the health effects of these irregular working hours [2, 3]. Education for flight crew members is an important component of these strategies, for which several educational programs have been developed. Although some of these programs have been studied, the effects and the optimal way to transfer the relevant knowledge remains largely unclear [4–8].

Literature on computerized health education shows that the content of advice should be tailored to the individual needs and should be applicable for all subgroups within a target population [9, 10]. Additionally, when translating the relevant flight crew related knowledge into practical advice, variables such as flight direction, flight duration, and number of time zones crossed should be taken into account [7]. Based on this knowledge, the MORE Energy intervention, aiming to reduce fatigue and improve health in airline pilots through easy obtainable and tailored advice, was developed [11]. The intervention provided participants with evidence-based and relevant fatigue-related advice using a mobile application (app), supported by a website with background information.

The usage of mobile health (mobile phone technologies in health care and public health) has expanded rapidly during the last decade [12]. Additionally, because the use of smartphones and tablets increased enormously, apps showed to have great potential for promoting health behaviour [13]. Evidence for these effects of mobile apps is still limited, however [14–17]. In addition, Blackman et al. [18] showed that mobile health studies scarcely report about key implementation factors, while this information is necessary to get more insight in the strength and weaknesses of the implementation of the intervention and to facilitate the interpretation of the results [19–21].

We, therefore, performed a process evaluation alongside our randomized controlled trial. The primary objective of this process evaluation was to assess the reach, dose delivered, compliance (dose received), fidelity, barriers and facilitators (context), and satisfaction of the MORE Energy intervention. The second objective was to investigate whether the MORE Energy intervention was associated with an improvement in relevant behavior of the participants by exploring the association between compliance to the intervention and the extent to which the pilots adhered, i.e. applied the advice in daily life. We also investigated if adherence was associated with the satisfaction of the pilots. The third objective was to investigate how the intervention suited the different subgroups within the target population. Therefore, outcome differences between pilots of the different age groups and haul types were analysed for the process evaluation items compliance and satisfaction.

## Methods

This process evaluation was carried out alongside the randomized controlled trial on the effectiveness of the MORE Energy intervention that aimed to reduce fatigue in airline pilots. The Medical Ethics Committee of the VU University Medical Center (Amsterdam, the Netherlands) assessed the study design and procedures, but according to Dutch law, this study proved to be exempt from a medical ethical review.

### Participants

The study population consisted of the pilots of a large internationally operating airline company. The pilots could participate in the study if they were not on sick leave for more than four weeks at the moment of recruitment and if they owned a smartphone or tablet with iOS (iPhone/iPad Operating System) or an Android operating system. After inclusion, the participants were equally randomized into an intervention group, and a control group which received a minimal intervention. Because this process evaluation addresses the MORE Energy intervention, we focus on the participants of the intervention group only.

### MORE Energy intervention

The MORE Energy intervention was developed systematically. First, a literature study was performed in order to gain insight in the latest scientific knowledge about optimal behaviour regarding disruption of the circadian rhythm and fatigue in flight crew. Next, focus groups were held to find out what medium and implementation strategy should be used to optimise compliance to the intervention. The focus groups made clear that the intervention should be easy available, appealing, and to be used by pilots of all ages and job types. Further, the advices should be made flight schedule specific and applicable for both short and long-haul pilots. To match the intervention with the legislation and the policy of the airline company, interviews with key management stakeholders were held as well.

Based on the focus groups and interviews, it was decided to develop a mobile application to transfer the advices to the target population. After the development of the MORE Energy app, it was extensively pre-tested by both pilots and researchers. Based on the results of this first evaluation, the intervention was optimised where necessary.

The MORE Energy app contained advices on optimal light exposure, sleep, nutrition, and physical activity, tailored to relevant flight (e.g. flight direction, departure and return time, number of time zones crossed) as well as to personal characteristics (e.g. job type, chronotype). The users could choose to consult background information in the glossary menu and the app guided them to a website to read more, or to view and listen to video and audio files concerning the different topics. Participants were encouraged to consider the advices on the app by means of two types of reminders: timed alerts (when the user did not use the app for longer than three weeks) and geofencing alerts (when the user arrived somewhere outside of the Netherlands, with a maximum of one alert per four days). Screenshots of the MORE Energy app can be seen in Additional file 1. Further details on the development, content and effect evaluation of the intervention have been published elsewhere [11, 22].

### Data collection

The process evaluation items were taken from the Steckler & Linnan framework [20]: reach, dose delivered, compliance (dose received), fidelity, barriers and facilitators (context), and satisfaction. Adherence, the extent to which the participants applied the advices in daily life, was measured as well. Table 1 presents an overview of the different items and the accompanying collection and processing of the data. The airline company provided data about the gender, age, job type, and haul type of all potential participants.

**Table 1:** Overview of the different process evaluation items

Items	Definition	Resources
Reach	Information on the number of participants (%) and their demographics, compared to the non-participants.	Information on all potential participants provided by the airline company.
Dose delivered	Total amount of intervention material provided to the participants (%).	The number of participants that was sent an email with instructions and login details.
Compliance (dose received)	Measured consultation of the tailored advice.	Objective: user authentication through the CMS (app) and Google Analytics (website). Subjective: online questionnaire.
Fidelity	Information on all changes, updates, and revisions that happened with the app during the intervention period. Calculated as the weighted average of the percentage of weeks the different components of the intervention were delivered as intended.	Log.
Satisfaction	Participants’ appreciation of the intervention and their opinion on its effectiveness (1–10).	Online questionnaire.
Barriers and facilitators (context)	Barriers and facilitators of the intervention, experienced by both the researchers and the participants.	Researchers: log. Participants: online questionnaire.
Adherence	The extent to which participants applied the MORE Energy advices in daily life.	Online questionnaire.

#### Reach

Reach is defined as the proportion and representativeness of the intervention group participants in the study, compared to the total group of potential participants [19]. Reach was determined by comparing the following characteristics between the intervention group participants and the airline pilots that did not participate: gender, age, job type, and haul type.

#### Dose delivered

Dose delivered is considered as the total amount of intervention material provided to the participants. In this study, the dose delivered was defined as the total number of participants that was sent an email containing instructions and login details to access the intervention material.

#### Compliance (dose received)

Compliance is the dose that is received, and refers to the extent to which participants actively engaged with the intervention. In our study, it was objectively measured through the Control Management System (CMS) of the application. This system stored the number of advices per week requested by each participant through user authentication. Likewise, we used a web-analytic tool (Google Analytics) to register and store the total number of page views per participant to the website of the project.

The registered number of app advices of four participants proved to be more than 200. Because this was most certainly due to malfunctioning of the CMS, the registered data of these participants was excluded from the objective compliance analyses.

The participants were also asked how often they had consulted the advices during the intervention (almost always, sometimes, only a few times, or never) in the online questionnaire at six months after baseline. Further, participants were asked which type of advice they had predominately used (advice regarding preparation for departure, regarding layover, or regarding the return home) and which topics they had consulted the most (exposure to light, sleep, nutrition, or physical activity).

#### Fidelity

Fidelity is defined as the extent to which the intervention program was implemented as planned, representing the quality and the integrity of the implementation [19]. Therefore, all changes, updates, and revisions of the app and website that occurred during the intervention period were kept in a log. Fidelity was calculated as the weighted average of the percentage of weeks of the total intervention period that the different components of the intervention were delivered as intended. As the advice delivered through the app was considered the main component of the intervention, this was given the largest weight, whereas the remaining four components were weighted equally:

- Access (installation, login, offline functionality): 15 %
- Backend (synchronisation of content and flight schedules): 15 %
- Advice (tailoring algorithm and glossary): 40 %
- Reminders (functioning of push alerts): 15 %
- Website with background information (access, functionality): 15 %

#### Satisfaction

The satisfaction with the intervention was assessed through the online questionnaire at six months after baseline. First, the participants were asked to give an overall grade for MORE Energy (1 to 10). Next, they were asked to rate four statements about the usability of the intervention on a 5-point Likert scale ranging from ‘disagree’ to ‘agree’. Additionally, participants were asked if they would recommend the MORE Energy application to their colleagues, and to appreciate the effectiveness of the intervention through rating three statements on perceived effectiveness on the 5-point Likert scale.

#### Barriers and facilitators (context)

Context refers to “the larger physical, social, and political environment that either directly or indirectly affects an intervention program” [20]. Possible context factors that affected the intervention were registered in a log. We also asked the participants which barriers or facilitators they had experienced. First, participants were asked if they would recommend the MORE Energy application to colleagues who did not have access to it yet. If they answered ‘no’, they were asked why they held that opinion. Next, participants were asked what their reasons were not to consult the advices more often (content already known, no need for further consultation, technical problems, lack of usability, or another reason).

#### Adherence

The extent to which participants applied the MORE Energy advices in daily life was assessed through asking the participants to rate the statement “After reading the advices, I actually applied them as well.” on a 5-point Likert scale ranging from 1 ‘disagree’ to 5 ‘agree’.

### Data analysis

Regarding the first objective, descriptive analyses were performed. Differences (gender, age, job type, and haul type) between participants and non-participants were analysed with t-tests for independent samples and Chi-square tests.

For the second objective, associations between compliance and the extent to which the participants applied the advice in daily life (adherence) were analysed by calculating Spearman's (rho) correlation coefficients. Regarding the objective compliance, participants were divided into four groups of equal size related to the amount of compliance. Furthermore, a linear regression analysis was performed to explore the association between the level of adherence (independent variable) and satisfaction with the intervention (dependent variable). Participants who indicated not to have applied the advices in daily life, or who had a neutral opinion towards this question, were used as one reference category in the analysis.

To answer the third objective, outcomes for the different age groups (<35, ≥35- < 45, ≥45) and haul types (short-haul vs. long-haul) on compliance and satisfaction were analysed with t-tests for independent samples, Chi-square tests and one-way ANOVAs. Participants who flew to intercontinental destinations were considered long-haul pilots, while participants who only flew to European destinations were considered short-haul pilots.

A two-tailed significance level of *p* < 0.05 was considered to be statistically significant in all analyses. Analyses were conducted with the Statistical Package for Social Sciences (SPSS), version 20.0.

## Results

### Reach

A total of 2222 potential participants were made aware of the project by means of a publicity campaign through the airline company, after which they received an email with a link to the baseline questionnaire. From this group, 522 (23 %) pilots agreed to participate. A total of 20 pilots (<1 %) did not meet the inclusion criteria. Of the remaining 502 pilots, 251 were randomized into the intervention group, and 251 into the control group. At the end of the intervention period, 148 (59 %) of the participants of the intervention group completed the process evaluation questionnaire. Baseline characteristics of the participants and the non-participants are shown in Table 2. Between the intervention and control group, no significant differences were present. The intervention group participants proved to be significantly younger than the non-participants, and the percentage of females was higher.

**Table 2:** Reach of the MORE Energy intervention; characteristics of the participants and the non-participants

Reach characteristics	Category	Intervention group (*n* = 251)	Control group (*n* = 251)	Non-participants (*n* = 1720)
Age in years mean (SD)		41.0 (8.0)*	40,7 (8,7)	42.5 (8.3)
Age group n (%)	<35	60 (23.9)*	66 (26.3)	313 (18.2)
	≥35- < 45	98 (39.0)*	89 (35.5)	652 (37.9)
	≥45	93 (37.1)*	96 (38.2)	755 (43.9)
Female n (%)		21 (8.4)*	13 (5,2)	65 (3.8)
Job type n (%)	Captain	111 (44.2)	113 (45,0)	750 (43.6)
	First Officer	97 (38.7)	96 (38,2)	706 (41.0)
	Second Officer	43 (17.1)	42 (16,7)	264 (15.3)
Haul type n (%)	Long-haul	179 (71.3)	179 (71,3)	1287 (74.8)
	Short-haul	72 (28.7)	72 (28,7)	433 (25.2)

* Significant difference between the participants and non-participants (*p* < 0.05)

### Dose delivered

We sent emails containing the login details and instructions to 251 participants. One email was bounced because the email address proved not to exist. After three months, one other participant reported to have not received the instruction email. The dose delivered therefore was determined to be 99 % (249/251).

### Compliance (dose received)

#### Objective compliance

It was registered that during the intervention period, 54 of the 251 participants never consulted any advice on the app. Two of these participants indicated that they wanted to drop out of the study. Five of them only consulted the website with the background information. The remaining 47 participants either did not download or use the app or website after receiving the instructions, did not receive or read the email with the instructions, or only used the glossary section of the app (consultation of the glossary was not registered by the CMS).

During the six month intervention period, 68 (27 %) participants consulted the advices during one week only, 54 (22 %) consulted them during two weeks, and 32 (13 %) consulted them during three weeks. A total of 43 (17 %) participants consulted the advices on the app during four weeks or more. In total, 1677 advices were requested. The mean number of requested advices per participant was 6.8 (SD 14.0), while the median was 3 advices per participant. If the data of the four participants with outliers was included, the mean number of requested advices would have been 12.6 (SD 55.3).

The CMS registered that the advices regarding the preparation for departure from home were requested the most (49 %). The advices concerned with time spent during layover (23 %) and the advices about the preparation for the return flight and arrival home (27 %) were consulted less often.

In total, 32 (13 %) participants went to the website with background information. The mean number of page views of these participants was 9.2 (SD 8.6). It was determined that 27 (11 %) participants used both the app and the website. Most of the participants did use the app but never logged on to the website (68 %).

#### Subjective compliance

Of the 148 participants that answered the process evaluation questionnaire, 62 (42 %) indicated they had never really used the MORE Energy advices, 39 participants (26 %) indicated that they had used the advices a few times, and 46 (31 %) reported to have used the advices occasionally. One participant reported to have used the advices before and during every flight. Next, of the 86 participants that had used the advices, most of them indicated that they had consulted the advices regarding layovers (70 %). Next, 49 % indicated to have consulted the advices regarding the return home, and 36 % indicated to have consulted the advices concerned with preparation before departure. Further, most of these participants pointed out that they consulted the advices concerned with sleeping behaviour (62 %) and nutrition (72 %). The advices regarding exposure to light (28 %) and physical activity (23 %) were consulted less often.

### Fidelity

Through the six month intervention period the implementation of the intervention was predominantly affected by the following (a detailed explanation of all bugs can be found in Additional file 2)

- After two weeks the second version of app became available for iOS: after downloading the update, participants could no longer consult all types of advices and iPhone5 users could not get access to the updated version of the app.
- After two months the third version of app became available for iOS to solve the problems above:
  - ∘ This version could not be installed automatically. Participants had to delete the previous version of the app before being able to install the new one.
  - ∘ After installation of third version, reminder alerts malfunctioned. Researchers found out this problem had most probably occurred in the Android app from the start of the intervention as well.

The five components of the intervention were affected to a different extent. Table 3 shows the number of weeks the components could be delivered as intended, and the calculation of the different fidelity scores. The weighted total fidelity score of the intervention was 67 %.

**Table 3:** Fidelity score calculation for the 26-week intervention period

Component		Weight factor	Weeks delivered as intended	Fidelity score
App	Access	15 %	16	62 %
	Backend	15 %	25	96 %
	Advice	40 %	19	73 %
	Reminders	15 %	0	0 %
Website	Access	15 %	25	96 %
Total fidelity score				67 %

### Satisfaction

**Fig. 1:**
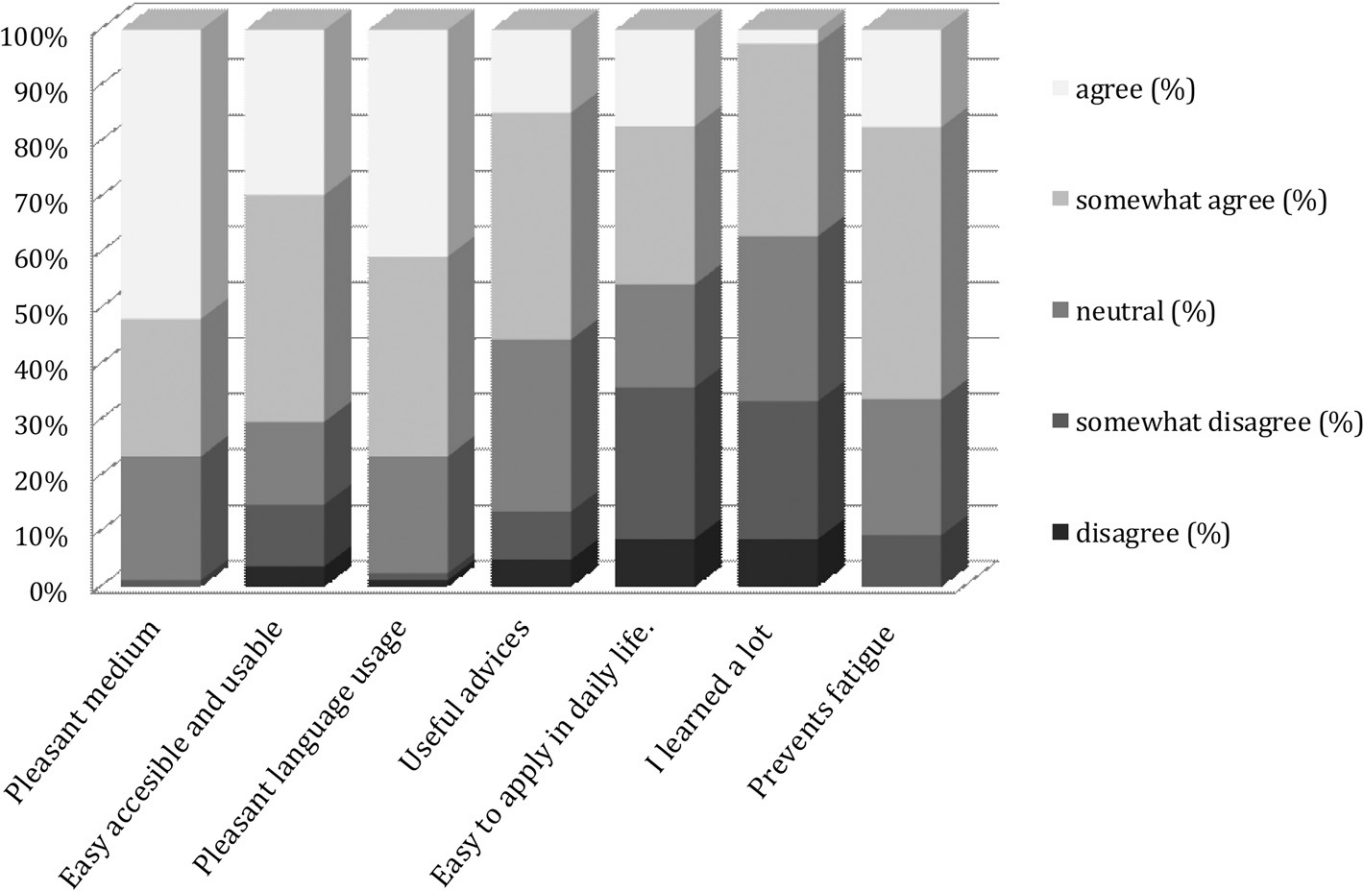
Perceived satisfaction with the MORE Energy smartphone application

Figure 1 shows the satisfaction of the participants who completed the process evaluation questions and indicated that they had used the advices of the MORE Energy app. The participants were satisfied with an app as the medium to transfer the advices (>75 % agreed) but less than half of the participants found that the advices given were easy to apply in their daily life (46 %). 56 % of the participants indicated that the MORE Energy advices were useful.

More than 65 % of the participants that used the advices thought that the MORE Energy intervention could prevent fatigue and improve fitness. Further, 78 % indicated that they would recommend the MORE Energy advices to their colleagues (data not shown). On average, the participants rated the MORE Energy intervention with a 6.4 (SD 1.6).

### Barriers and facilitators (context)

The 86 pilots that indicated to have used the advices were asked what their reasons were not to consult the advices more often. The most selected reason was that the content of the advice was already known to them (58 %). Furthermore, 44 % of the participants indicated that they did not need to consult the advices anymore after a few times. Less selected reasons were technical problems with the app (12 %) and lack of usability (7 %). Out of the additional reasons reported by 24 participants, three main themes could be composed. First, nine participants reported that the advices could not be applied in daily life because that would conflict with their social obligations, both at home and during duty. Predominantly participants with young children and short-haul pilots pointed out this problem. Four participants indicated that they had simply forgotten to consult the app, or that they did not receive an alert to remind them. Similar barriers were addressed by the 19 participants who reported that they would not recommend the MORE Energy app to their colleagues; the advices were too common or not innovative enough (*n* = 5), the advices were not applicable in daily life (*n* = 4), the content was already known (*n* = 3), or the app had too much technical problems (*n* = 2).

### Association between compliance, adherence, and satisfaction

Of the participants indicating to have used the advices during the intervention period, 47 (55 %) somewhat agreed, and 14 (16 %) agreed with the adherence statement that they applied the advices after consulting them. Further, 25 (29 %) participants somewhat disagreed or had a ‘neutral’ opinion towards the statement.

Spearman’s rho correlation between the objectively and subjectively measured compliance and adherence was 0.04 (*p* = 0.71) and 0.30 (*p* = 0.004), respectively.

Table 4 shows that participants who indicated to have applied the advices after consulting them rated the intervention significantly higher compared to the participants of the reference category. The participants who somewhat agreed with the adherence statement, rated the intervention significantly higher as well (*p* ≤ 0.001).

**Table 4:** Linear regression analysis results for adherence and satisfaction with the intervention

	β	*p*-value	95%CI	
			Lower	Upper
Agree (*n* = 14)	2.42	0.000	1.41	3.42
Somewhat agree (*n* = 47)	1.40	0.000	0.68	2.11
Neutral or disagree (*n* = 25)	Reference			

*95%CI* 95 % Confidence Interval

### Subgroup differences

#### Compliance

The objective compliance of the subgroups was comparable with compliance in the total group of participants. As can be seen in Table 5, differences between the groups were small. Because layover advices were not available for short-haul pilots, their registered number of consultations of this type of advice was close to zero.

**Table 5:** Compliance scores within the subgroups

Compliance															
Subgroup	Objective (*n* = 247)					Subjective (*n* = 86)									
	Mean number of advices (SD)	Median	Type of advice (% of advices)			% using the advices			Type of advice (% of users)			Subject of advice (% of users)			
			Home	During layover	Return	Never	A few times	Sometime/always	Home	During layover	Return	Light	Sleep	Nutr.	Phys. act.
<35	5.65 (10.76)	2.00	51 %	25 %	25 %	53 %	26 %	21 %	28 %	78 %	56 %	17 %	78 %	83 %	11 %*
≥35- < 45	6.34 (13.21)	3.00	52 %	18 %	29 %	39 %	30 %	32 %	43 %	63 %	46 %	34 %	57 %	80 %	43 %*
≥45	7.98 (16.36)	2.00	46 %	28 %	27 %	38 %	23 %	40 %	33 %	73 %	48 %	27 %	58 %	58 %	9 %*
Short-haul	7.42 (14.34)	2.00	64 %	1 %	35 %	44 %	28 %	28 %	50 %	68 %	36 %	32 %	59 %	91 %*	36 %
Long-haul	6.53 (13.84)	3.00	42 %	34 %	24 %	41 %	26 %	33 %	31 %	70 %	53 %	27 %	63 %	66 %*	19 %

* Significant difference (*p* < 0.05)

Results on subjective compliance show that 53 % of the pilots younger than 35 years indicated to have never consulted the advices. Forty percent of the oldest group of pilots indicated that they used the advices sometimes or always. All age groups indicated they consulted the advices about the time spent during layover the most (63 to 78 %), followed by advices concerned with the return flight (46 to 56 %), and the advices before departure from home (28 to 43 %). With regard to the content of the advices, the youngest group of pilots was more concerned with advice regarding sleep (78 % vs. 57/58 %, NS), while the oldest group was less concerned with advice regarding nutrition compared to their colleagues (58 % vs. 80/83 %, *p* = 0.06). Further, significantly more 35 to 45 year old pilots were interested in advice regarding physical activity than their younger and older colleagues (43 % vs. 9/11 %, *p* = 0.002).

Although layover advice was not available for short-haul pilots, subjective compliance results show that both groups indicated to have consulted this type of advice the most. Next, 50 % of the short-haul pilots consulted the advices with regard to departure from home, while 53 % of the long-haul pilots consulted the advices regarding the return flight. Advice regarding nutrition was consulted significantly more by short-haul compared to long-haul pilots (91 % vs. 66 %, *p* = 0.02).

#### Satisfaction

No significant differences in satisfaction were present between the subgroups (Table 6). The youngest group of pilots showed the lowest percentage that agreed that the app was accessible and usable (61 %). However, this group showed the highest percentage that reported the advice to be easy to apply (56 %) and that indicated to have learned from the intervention (44 %). Of the oldest group of pilots, 40 % indicated that the advices were easy to apply, and 33 % indicated that they learned a lot. Still, 85 % of the oldest group of pilots would recommend the intervention to their colleagues.

**Table 6:** Satisfaction scores within the subgroups

Subgroup	Rating	Satisfaction (agree)							
	Mean grade (SD)	Pleasant medium	Accesible and usable	Pleasant language	Useful advices	Easy to apply	Learned a lot	Prevents fatigue	Recommend to colleagues
<35	6.2 (1.5)	83 %	61 %	83 %	61 %	56 %	44 %	61 %	72 %
≥35- < 45	6.5 (1.5)	67 %	73 %	73 %	45 %	45 %	36 %	63 %	74 %
≥45	6.5 (1.9)	83 %	73 %	77 %	63 %	40 %	33 %	62 %	85 %
Short-haul	6.1 (1.9)	75 %	75 %	85 %	40 %	55 %	30 %	70 %	70 %
Long-haul	6.5 (1.5)	77 %	69 %	74 %	61 %	43 %	39 %	70 %	87 %

Comparing the two haul types, it can be seen that 55 % of the short-haul pilots reported that the advices were easy to apply, compared to 43 % of the long-haul pilots. However, 40 % of the short-haul pilots considered the advices also useful, compared to 61 % of the long-haul pilots (*p* = 0.06). Further, 70 % of the short-haul pilots would recommend the advices to their colleagues, while 87 % of the long-haul pilots would do that (*p* = 0.06).

## Discussion

### Main findings

The primary objective of this process evaluation was to assess the reach, dose delivered, compliance, fidelity, context, and satisfaction of the MORE Energy intervention. The reach among the source population was 22 %. This percentage is quite high compared to the 1.5 to 8 % reach published in other mobile health studies [16, 23, 24], and compared to more conventional studies promoting health behaviour at worksites [25].

The participating pilots were significantly younger compared to the non-participants, possibly because younger pilots are more familiar with mobile apps. Female pilots were also overrepresented, possibly caused by the fact that women tend to exhibit more active information-seeking behaviour and are more likely to participate in scientific studies than men in general [26, 27].

It was shown that the dose delivered and initial compliance was high. Of the participants, only 19 % never used any advice on either the app or the website. It is even possible that some of these participants never received the email containing the instructions and login details, because of for instance a strict junk-email filter. However, during the intervention period, only one participant reported not to have received the instruction email.

The compliance during the whole intervention period was rather low with 17 % of the participants consulting the advices on the app for more than four weeks during six months. Technical problems with some components of the app, as could be seen in the calculation of the fidelity score, might have contributed to this. The fidelity score of 67 % is difficult to interpret since we are the first mobile health study to calculate such a score. Moreover, a mobile health intervention review showed that only 13 % of the published mobile health studies on physical activity promotion reported fidelity information whatsoever [18].

Our results showed a distinction between the objectively and subjectively measured compliance regarding the type of advices. Despite the registered data displayed that the advice concerned with departure from home was consulted most often, the participants themselves indicated that they used the advice concerned with layovers the most. It might be possible that the participants thought that they were asked *when* they had used the advice most often. This would also explain the finding that short-haul pilots indicated to have used the layover advice the most although that type of advice was not available for them; they might have predominately considered the advice regarding their next flight at the end of a duty day (i.e. during their layover).

Despite the moderate compliance scores, a majority of the users (65 %) was convinced that the intervention was able to fulfill its purpose, preventing fatigue and improving health of pilots. Also, 78 % of the participants would recommend the intervention to their colleagues. In this perspective, the 6.4 (range 1 to 10) appreciation score for the MORE Energy intervention as a whole, is somewhat low. On the other hand, a majority of the pilots (54 %) did not agree with the statement that the advices were easy to apply in daily life. This lack of applicability of the flight schedule specific advices was also shown in the barriers and facilitators section. Participants indicated that applying the advices would conflict with their social life at home (e.g. young children) or during duty (e.g. habits during layovers). Furthermore, the correlation between registered compliance and adherence proved to be very low (*r* = 0.041). This might indicate that although participants were interested to see what advice the app would provide regarding their upcoming flight schedule, the content did not pursue them to change their behavior. However, once applied, the advices might have been useful: participants that did indicate to have applied the advices in daily life rated the intervention significantly higher compared to the participants who did not apply them.

Another objective of this process evaluation was to assess whether there was a difference in compliance and satisfaction outcomes between participants of the two haul types and the three age groups present in the population. The results showed that the differences in both objective and subjective compliance between the age groups were small. The oldest participants tended to be the most critical regarding the applicability of the advices. Possibly, these pilots were unwilling to give up the patterns and habits which they developed throughout their career. This was already mentioned during the focus group interviews before the development of the intervention: it would be hard to alter the (social) patterns of the more experienced colleagues. The apparent contradicting high percentage of the oldest group of pilots (85 %) that would recommend MORE Energy to their colleagues may be in accordance: these pilots do not need the advice for themselves but they think it might be useful for their less experienced colleagues.

Regarding the participants of the two haul types, no significant differences in objective compliance existed either. The relatively large number of short-haul pilots that participated in the study did find the advices easy to apply, but found them not very useful. Consequently, short-haul pilots tended to be less satisfied with the intervention compared to the long-haul pilots. Although the operations of the two haul types differ substantially, short-haul schedules can trigger fatigue as much as intercontinental schedules [28]. However, most of the scientific knowledge and practical advice available concerns the disruption of the circadian rhythm and is mostly applicable for long-haul pilots. Consequently, the content of our specific advice regarding short-haul schedules was less extensive. The short-haul pilots participating in the test phase of the intervention noticed this already. Although we reconsidered and extended the short-haul advices afterwards, the outcomes of our process evaluation indicate that this elaboration was probably not sufficient.

### Strength and limitations

This process evaluation is one of the first evaluating an mobile health intervention promoting health behaviour. One of the major strengths is that we used a combination of objective and self-reported data to evaluate the implementation of the MORE Energy intervention. The compliance with both the app and the website was registered through user authentication, which is more reliable than self-reported information [29]. Nevertheless, most probably due to malfunctioning of the CMS, data of four participants had to be excluded from the analyses. We did use self-reported data as well, to gather more detailed information on subject specific compliance, adherence and appreciation.

We achieved a reach of 22 %. Although this is quite high compared to participation rates published in other mobile health studies, the generalizability of the results might be hampered due to selection effects. It is possible that the non-participating pilots did not possess an iOS or Android smartphone or tablet, or were not sufficiently familiar with mobile apps. The comparison of the participants with the non-participants also showed that participants were significantly younger, and that a relatively large number of female pilots participated.

Another limitation of this study is that only 59 % of the participants filled out the process evaluation questionnaire. Therefore, selection bias may have occurred, which challenges the reliability and validity of the outcomes. Another limitation of the study is that we could not measure all activities of the users of the mobile app. The number and types of requested advices were registered, but consultation of the background information and time spent on the app was not.

The MORE Energy intervention aimed to improve relevant behaviour among airline pilots. Although we asked participants whether they had applied the advices in daily life, their actual change in behaviour could not be objectively measured. Therefore, to maximize the interpretation of our results, it would have been useful if the app had used more built-in features of smartphones to measure behaviour (e.g. timing of sleep using the motion sensor of the mobile device) [13].

### Implications for research and practice

The reach of this study shows that airline pilots are willing to participate in an intervention using an app to promote health behaviour. Although guidelines have been proposed to improve the way the outcomes of mobile health interventions are reported [21], qualitative process evaluations publications are necessary as well in order to provide more information about the implementation and working mechanisms of these kind of interventions [18].

High compliance is important for the success of any intervention intending to modify behaviour, but especially so in web-based interventions since there is no direct contact with the participants [14]. Because we found that compliance dropped since the start of the intervention, similar studies should put more effort into keeping participants involved throughout the intervention period. Although technical problems were not mentioned as main reasons for non-compliance, time consuming update installations and the resulting loss in functionality, might have been of influence. One of the updates during the intervention period caused the malfunctioning of the reminder alerts. This might have led to a decrease in compliance since well-timed and adequate prompts can be effectively used in mobile health intervention studies. Other possibilities that could keep users engaged involve altering and updating the content of the intervention material, providing personal feedback, and introducing goal setting [12].

Our process evaluation gives insight into the different aspects involved in the implementation of the MORE Energy intervention, and will help to improve the interpretation of the results of the trial. The outcomes of the different items showed which parts of the intervention should be improved before offering the intervention to all flight crew throughout the airline company. First, the app should pursue users more to change relevant behavior, despite the consequences for their social responsibilities. Further, the content of the advice should be better applicable for both experienced and inexperienced employees, involved in both short and long-haul schedules. The airline company involved can assist the subsequent implementation of the improved app by integrating it with flight crew scheduling, by giving sustained attention to the topic within the present flight crew members, and by introducing the tool to newly hired employees [7]. After the improvement of the MORE Energy intervention, it might be transformed into a ‘white label’ tool in order to make it possible to adapt and implement it as a fatigue management tool for flight crew members or shift workers in other companies as well.

## Conclusions

The process evaluation of MORE Energy showed that this mobile health intervention was well received, resulting in an adequate reach and a high dose delivered. Although more than 80 % of the participants did use the intervention, most of them were not compliant throughout the intervention period. The intervention could not be delivered as intended and perceived satisfaction was moderate. Further, the combination of compliance and satisfaction scores indicates that the content and applicability of the advices should be improved to appeal all subgroups first, before making the app available for the other flight crew members within the airline company. After this improvement, MORE Energy might be adapted and implemented as a fatigue management tool for other employees and companies as well.

## Additional files

Additional file 1: Screenshots of the mobile application used during the MORE Energy intervention. (PDF 1072 kb) Additional file 2: Detailed explanation of all bugs that occured during the intervention period. (DOCX 16 kb)

## Abbreviations

95%CI: 95 % Confidence Interval
App: Application
CMS: Control Management System
iOS: Operating System for iPhone or iPad
SD: Standard Deviation
