# Supplementary material for: Process evaluation of a tailored mobile health intervention aiming to reduce fatigue in airline pilots
Source: BMC Public Health. 2016 Aug 26;16(1):894. doi: 10.1186/s12889-016-3572-1 (PMC5002199; doi:10.1186/s12889-016-3572-1)

MORE Energy app

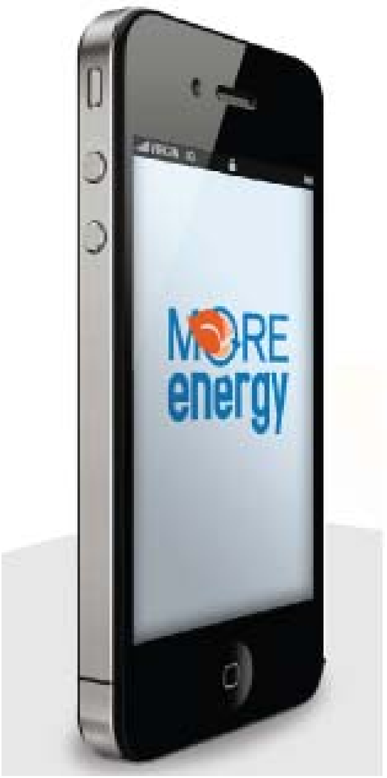

Available in appstores

(iTunes and Google Play)

1. Login & select flight

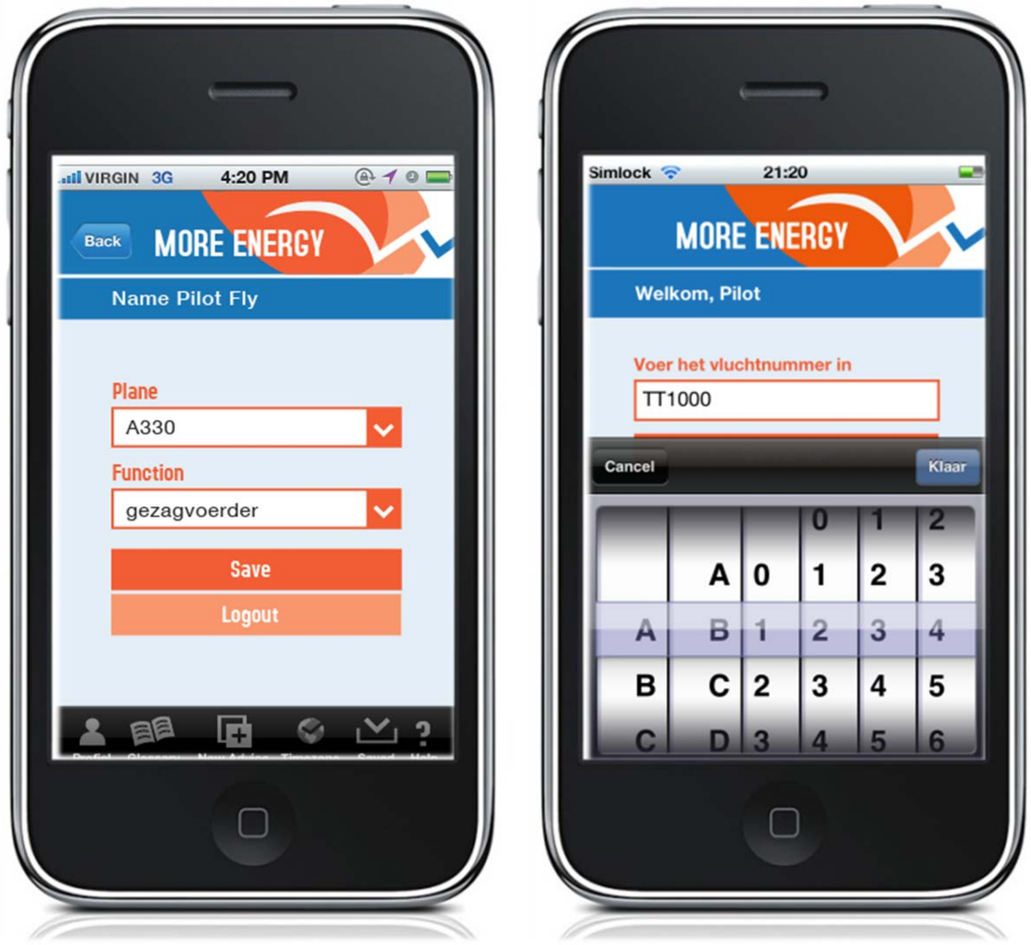

2. Select specific advice

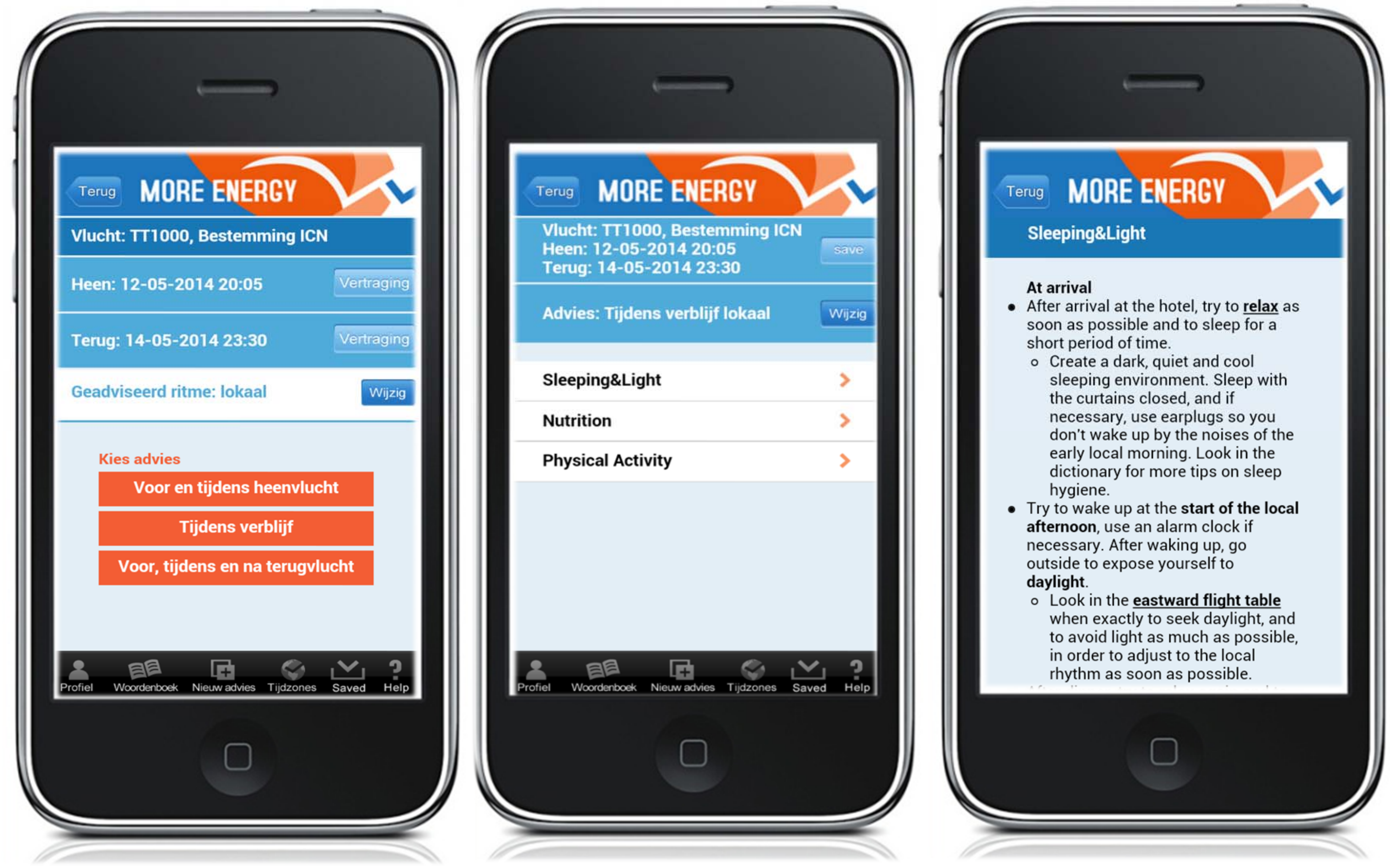

3. Glossary menu

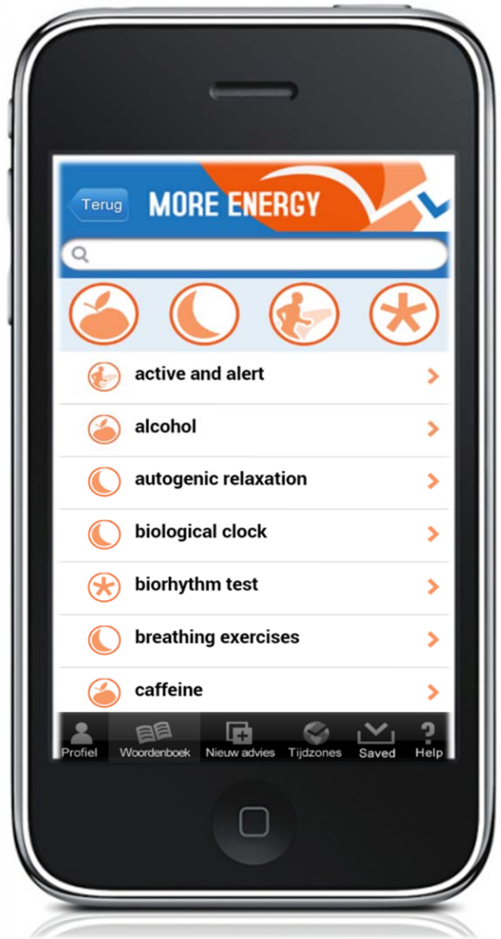

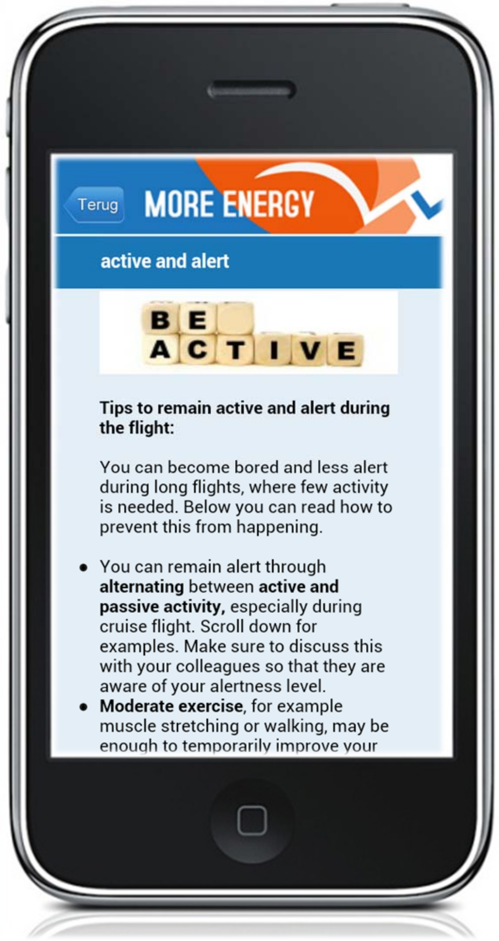

4. Link to project website

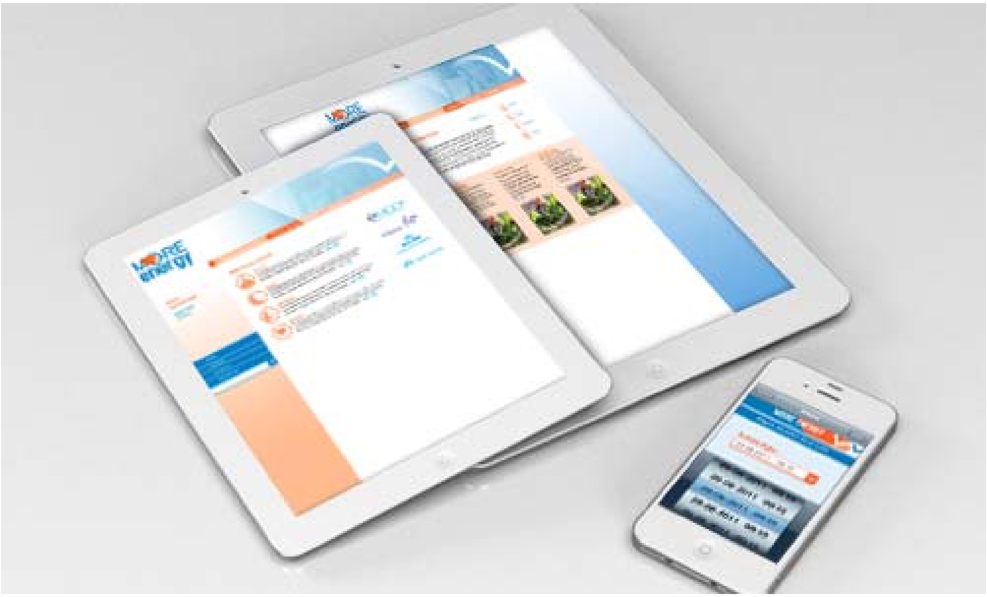

Supplement: Additional file 1: — Screenshots of the mobile application used during the MORE Energy intervention. (PDF 1072 kb) [file 12889_2016_3572_MOESM1_ESM.pdf]
