# Supplementary material for: Process evaluation of a tailored mobile health intervention aiming to reduce fatigue in airline pilots
Source: BMC Public Health. 2016 Aug 26;16(1):894. doi: 10.1186/s12889-016-3572-1 (PMC5002199; doi:10.1186/s12889-016-3572-1)
Supplement: Additional file 2: — Detailed explanation of all bugs that occured during the intervention period. (DOCX 16 kb) [file 12889_2016_3572_MOESM2_ESM.docx]

Additional file 2. Bugs during the MORE Energy intervention period

- Every month, the researchers uploaded the new flight schedules of the airline company into the control management system (CMS) of the application. The app was designed to synchronise with the CMS automatically so that the users could always consult the advices belonging to the latest flight schedules. During the whole intervention period three users reported that this synchronisation took very long (more than 15 minutes). The only way to resolve this problem proved to be reinstalling the app as a whole. Because the developers of the app could not reproduce this specific problem, it could not be resolved before the end of the intervention period.
- In the second week of the intervention period, an update of the app (version 2) was uploaded in the Apple app store to make the app compatible with the new operation system, iOS6. After the update, several users started to report two problems.
  - Advices concerning the layover after ‘neutral’ flights (flights without time zone crossings) were not shown anymore.
  - Users who used the app on an iPhone5, reported that they could not open the app on their device at all.
- Therefore, the researchers instructed the app developers to come up with a new version in which these problems were resolved. After two months of the intervention period (February 2013), this third version of the MORE Energy app was made available in the Apple app store.
- In the weeks that followed, however, it became clear that the new version of the app was not compatible with the previous version. Nine participants reported that, whenever opening version 3, an error forced the app to close down. It turned out that the only way to solve this problem was to delete the whole app, and to subsequently download and install the new version again. Three weeks after the upload of version 3, all participants of the intervention group were sent an email instructing them to delete the app before installing version 3 (again).
- At the beginning of April 2013 the researchers themselves noticed that on Apple devices, they did not receive any more push alerts, which were designed to improve app usage. Afterwards, it proved very difficult and time consuming for the app developer to find out what caused this problem. It was not before two weeks before the end of the intervention period that the cause of the problem was detected. Consequently, it is assumed that none of the participants with an Apple device has received any kind of reminder or alert during the last three months of the intervention period. The process evaluation showed that the majority (95%) of the participants that consulted the advices were owners of an Apple device.
- Furthermore, it is possible that the participants with an Android device did not receive all reminders either. Although not reported by participants, the researchers who possessed an Android device, experienced that the geofencing reminders, the alerts which were given outside the Netherlands, with a maximum of one alert per four days, were malfunctioning through the whole intervention period (e.g. they received a reminder when they were at home, and did not receive anything when abroad).
- Other matters that occurred during the intervention period:
  - Because of a wrong setting in a flight schedule upload, the app provided the users with wrong advices for two days after 3.5 months of the intervention period.
  - After 5.5 months, the project website was offline for two days because of a hosting problem.
  - Three participants reported that the app did not work on their tablet with an older operating system. This was resolved after they updated their operating system.
